# Machine learning-enhanced stochastic uncertainty and sensitivity analysis of the ICRP human respiratory tract model for an inhaled radionuclide

**DOI:** 10.1088/1361-6498/ad7ec3

**Published:** 2024-10-16

**Authors:** Emmanuel Matey Mate-Kole, Sara C Howard, Ashley P Golden, Shaheen Azim Dewji

**Affiliations:** 1 Nuclear and Radiological Engineering and Medical Physics Programs, Georgia Institute of Technology, Atlanta, GA, United States of America; 2 Epidemiology and Exposure Science, Oak Ridge Associated Universities, Oak Ridge, TN, United States of America

**Keywords:** stochastic expansion, uncertainty and sensitivity analysis, biokinetic models, ICRP HRTM, machine learning

## Abstract

The International Commission on Radiological Protection (ICRP) has developed the reference Human Respiratory Tract Model (HRTM), detailed in ICRP Publications 66 and 130, to estimate the deposition and clearance of inhaled radionuclides. These models utilize reference anatomical and physiological parameters for particle deposition (PD). Biokinetic models further estimate retention and excretion of internalized particulates, aiding the derivation of inhalation dose coefficients (DC). This study aimed to assess variability in deterministic ^131^I biokinetic and dosimetry models through stochastic analysis using the updated HRTM from ICRP Publication 130. The complexities of the ICRP PD model were reconstructed into a new, independent computational model. Comparison with reference data for total PD fractions for reference worker, solely a nose breather, covering activity median aerodynamic diameters from 0.3 *μ*m to 20 *μ*m, showed a 1.04% relative and 0.7% absolute difference, demonstrating good agreement with ICRP deposition fractions. The deterministic DC module was reconstructed in Python and expanded for stochastic analysis, systematically expanding deposition components from HRTM and assigning probability distribution functions to uncertain parameters. These were integrated into an in-house stochastic radiological exposure dose calculator, utilizing latin hypercube sampling. A case of an occupational radionuclide intake was explored, in which biodistribution and committed effective DC (CEDC) were computed for ^131^I type F, considering a lognormal particle size distribution with a median of 5 *μ*m. Results showed the published ICRP reference CEDC marginally exceeds the 75th percentile of observed samples, with log-gamma distribution as the best-fit probability distribution. A Random Forest regression model with SHapley Additive exPlanations was employed for sensitivity analysis to predict feature importance. The analysis identified the HRTM particle transport rates scaling factor, followed by the aerodynamic deposition efficiency in the alveolar interstitial region as the most impactful parameters. This study offers a unique stochastic approach on inhaled particulate metabolism, enhancing radiation consequence management, medical countermeasures, and dose reconstruction for epidemiological studies.

## 1. Introduction

The discipline of internal dosimetry focuses on estimating radiation doses resulting from the distribution of radionuclides within bodily tissues and organs (Zanzonico 2000, Mate-Kole and Dewji 2024). Radionuclides can enter the body through various pathways, such as inhalation, ingestion, injection, or dermal absorption (Li 2018). Once inside the body, these radionuclides may be absorbed into the bloodstream through the respiratory or gastrointestinal tracts or directly absorbed into the bloodstream, where they are subsequently transported and distributed among different organs and tissues. Elimination routes for these radionuclides include urine, feces, exhalation, and sweating through the skin. Since direct measurement of radionuclide levels in specific organs of the human body is not feasible, internal dosimetry heavily relies on sophisticated mathematical models known as biokinetic models (Bertelli *et al* 1997). These models represent the dynamic processes of radionuclide uptake, retention, and clearance within biological systems over time.

Biokinetic models conceptualize the movement of radionuclides or contaminants into and out of the body as a system of interconnected ordinary differential equations (ODEs) (Li 2018). Organs and tissues described in the biokinetic models are termed source organs of radiation due to the continuous uptake of incorporated contaminants. Radionuclides in source organs may lead to the irradiation of nearby target organs or tissues, resulting in energy deposition. The energy deposited can result in biological effects, such as DNA alterations, cell damage, or the potential for cancer development. (ICRP 1991, Li 2018).

Within the context of inhalation exposure, the International Commission on Radiological Protection (ICRP) has developed reference models with deterministic quantities known as the Human Respiratory Tract Model (HRTM), which has undergone iterative refinement since its introduction in ICRP Publication 66 (ICRP 1994), with updates provided in ICRP Publication 130 (ICRP 2015). The HRTM relies on predefined reference structural, anatomical, and physiological parameters outlined by the ICRP. As delineated in ICRP Publication 130 (ICRP 2015), these parameters are fixed and recommended by the ICRP for dosimetric calculations, without considering uncertainties. These deterministic parameters are representative of a reference individual—an idealized person, for whom the equivalent doses to organs and tissues are calculated by averaging the corresponding doses of a reference male and female with anatomical and physiological characteristics defined by ICRP for radiological protection (ICRP 2002, 2015). While this methodology serves as a valuable tool for planned, prospective radiation protection strategies aimed at the general population or reference occupational cohort, it is important to note that it is reasonable to assume that few individuals within any exposed population will precisely align with the reference person (NCRP 1998).

While the conventional biokinetic and dosimetry models provided by the ICRP are appropriate for guiding predictive radiation protection measures, their dependence on predefined reference parameters introduces constraints when applied to consequence management scenarios, radiation and medical countermeasure applications, and epidemiological dose reconstruction in cohort studies. These scenarios extend beyond instances of widespread population exposure to radiological or nuclear sources, for which reference models are tailored, to include occupational, medical, specialized cohorts and environmental settings where non-standard conditions prevail. Within these diverse contexts, variations in individual anatomical, physiological, and metabolic characteristics, and uncertainties—define as the lack of knowledge of the central population value (Paquet 2022), exert considerable influence on dosimetric predictions and radiation response strategies.

In emergency response scenarios, various needs may emerge (Paquet 2022) necessitating consideration of diverse approaches such as stochastic effects, specific tissue responses, case-specific conditions and individual characteristics (e.g. bone marrow suppression, thyroid blocking, dehydration, accelerated respiratory activity, psycho-social impacts leading to behavioral changes, individuals with specialized health conditions like kidney disease, sex- and age-specific metabolism and physiology).

Prior studies have assessed the particle deposition (PD), clearance, and sensitivity within the parameters of the ICRP Publication 66 model using the LUng Dose Uncertainty Code (LUDUC) (Bolch *et al* 2001, 2003, Huston *et al* 2003, Farfán *et al* 2004). The purpose of LUDUC was to assess the effects of parameter uncertainties within the deposition and the clearance of inhaled particles on lung doses specifically from ^239^PuO_2_ and ^238^UO_2_/^238^U_3_O_8_ using the ICRP Publication 66 HRTM (ICRP 1994). The overarching goal of the study presented herein was to assess the variability in deterministic biokinetic/dosimetry models and better represent a stochastic consideration of radionuclide metabolism resulting from realistic source term intakes. Specifically, this study is aimed at conducting stochastic analysis of uncertain biokinetic parameters in the human respiratory tract through uncertainty and sensitivity analysis of an ^131^I (type F) inhalation scenario, thereby providing more accurate and robust estimates of inhalation dose coefficients (DC). The analysis considered factors such as radionuclide form, particle size distribution, morphology, solubility, and clearance, and utilized the ICRP Publication 130 HRTM. Radioactive iodine was chosen for this study due to its interaction characteristics and behavior when released into the environment; it can disperse rapidly in the air, but it can also combine with organic materials and move slowly through the environment (USEPA 2024). Iodine may be encountered in the industry in a variety of chemical and physical forms, including vapors and gases, organic compounds, and particulates (ICRP 2017). As a fission product, ^131^I is associated with nuclear power plant releases (DJurović *et al* 2016, Sulaiman *et al* 2018) or an improvised nuclear device (Marcus *et al* 2006, Chen and Tenforde 2010, Pan and Ungar 2012). Moreover, according to the U.S. Environmental Protection Agency (EPA), ^131^I is one of the two most commonly used radioisotopes, widely utilized in both diagnostic and therapeutic medical applications, and is considered to have the greatest environmental impact if released (NCRP 2019, USEPA 2024).

## 2. Methods

The methodological approach comprises three primary phases aimed at developing a mathematical and computational framework for stochastic biokinetic modeling based on source term intakes, specifically targeting inhalation DC of inhaled ^131^I with an activity median aerodynamic diameter (AMAD) of 5 *μ*m. As per the ICRP (2017), the recommended default type for iodine is F (fast clearing type), with the following assigned chemical matrices: sodium iodide, cesium chloride vector, silver iodide, and any form that is either unknown or known but lacks information regarding absorption from the respiratory tract. Accordingly, the type adopted in this study was type F.

The first phase of the study involved the development of a computational module in the Radiological Exposure Dose calculator (REDCAL) from the Radiological Engineering, Detection, and Dosimetry Laboratory at Georgia Institute of Technology in the Python programming language for biokinetic and dosimetry evaluation, customizable for stochastic expansion. Models incorporated in this phase of the study included the ICRP PD model, the ICRP Publication 130 HRTM, and an element-specific systemic biokinetic model, for which iodine was selected in this study. Iodine biokinetics were coupled with radiation-weighted *S* coefficients, representing the mean absorbed radiation dose to a target organ and tissue. The next phase of the study entailed investigating uncertain parameters in the human respiratory tract applicable to the ICRP Publication 130 HRTM. The last phase of the study focused on the stochastic analysis of the biokinetic model from inhaled ^131^I source term through the ICRP Publication 130 HRTM incorporating random forest (RF) models—a machine learning technique (Pedregosa *et al* 2011)—coupled with SHapley Additive exPlanations (SHAP) (Lundberg *et al* 2020) for feature importance. The RF regression model utilized in this study is based on Gini Impurity (Pedregosa *et al* 2011), whereby feature importance is calculated based on how much each feature contributes to reducing impurity across all the decision trees. The RF model was coupled with SHAP to enhance transparency in interpretation by providing a comprehensive machine learning model interpretation. The stochastic expansion included computational implementation of latin hypercube sampling (LHS) (Dalbey *et al* 2021) technique for sampling uncertain parameters, propagation of uncertainty, and sensitivity analysis through the deterministic module, and characterization of distribution of the inhalation DC via Kolmogorov–Smirnov (K–S) (Massey 1951) test for goodness of fit.

### 2.1. Computational module for biokinetic and dosimetry evaluation

**Figure 1.**
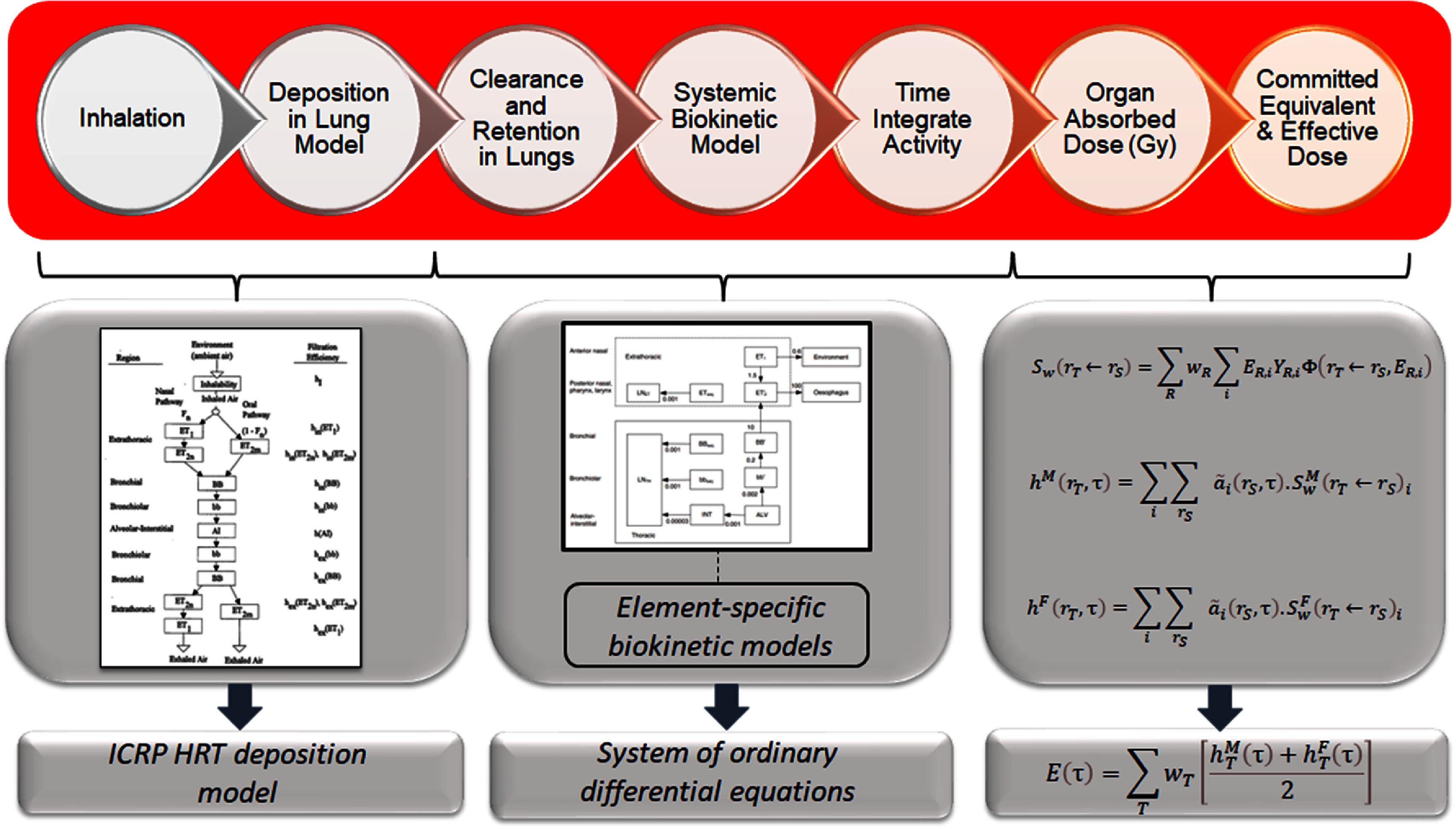
Inhalation dose coefficient (DC) computational framework. The first block from the left estimates the particle deposition in the respiratory tract based on the ICRP empirical model. Middle block computes the retention in the source organs and the first block from the right integrates the activity retained and the radiation weighted absorbed dose to estimate committed effective dose coefficient (CEDC). ${S_w}\left( {{r_T} \leftarrow {r_S}} \right)$ is the radiation weighted *S* coefficient quantifying the mean absorbed radiation dose, ${h^M}\left( {{r_T},\tau } \right)$ and ${h^F}\left( {{r_T},\tau } \right)$ are the committed equivalent DCs for tissues in the reference male and the reference female, and $E\left( \tau \right)$ is the CEDC. The figure for the filtration mechanism illustrated in the particle deposition block was reproduced with permission from the ICRP Publication 66, figure 8 (ICRP 1994), and the HRTM in the middle block was reproduced with permission from the ICRP Publication 130, figure 3.4 (ICRP 2015).

**Figure 2.**
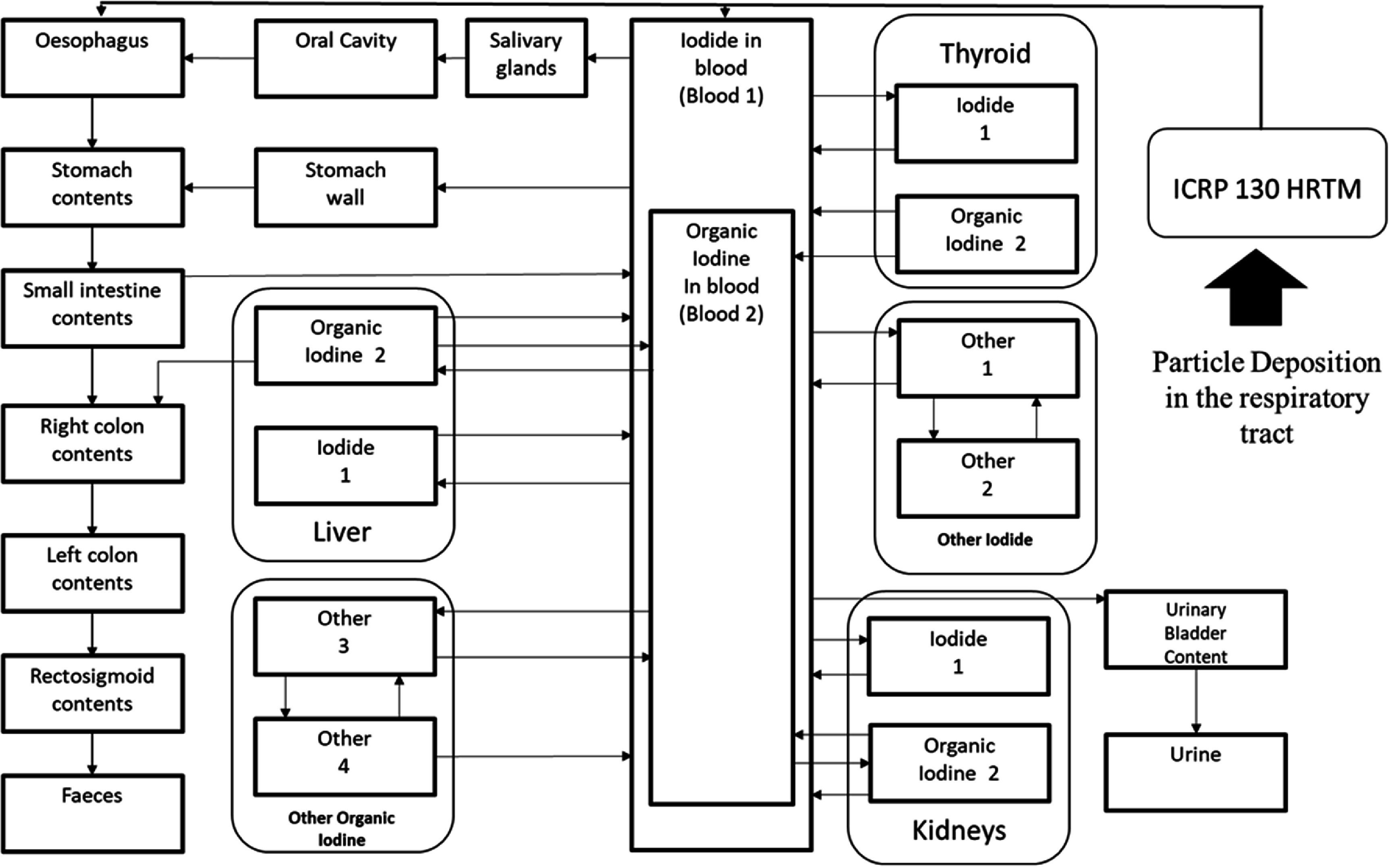
Coupled inhalation compartmental model for ^131^I. Arrows indicate a transfer of iodine from one organ/tissue to another with a designated transfer coefficient in units per day (d^−1^). The systemic model for iodine was reproduced with permission from the ICRP Publication 137, figure 5.2 (ICRP 2017).

**Figure 3.**
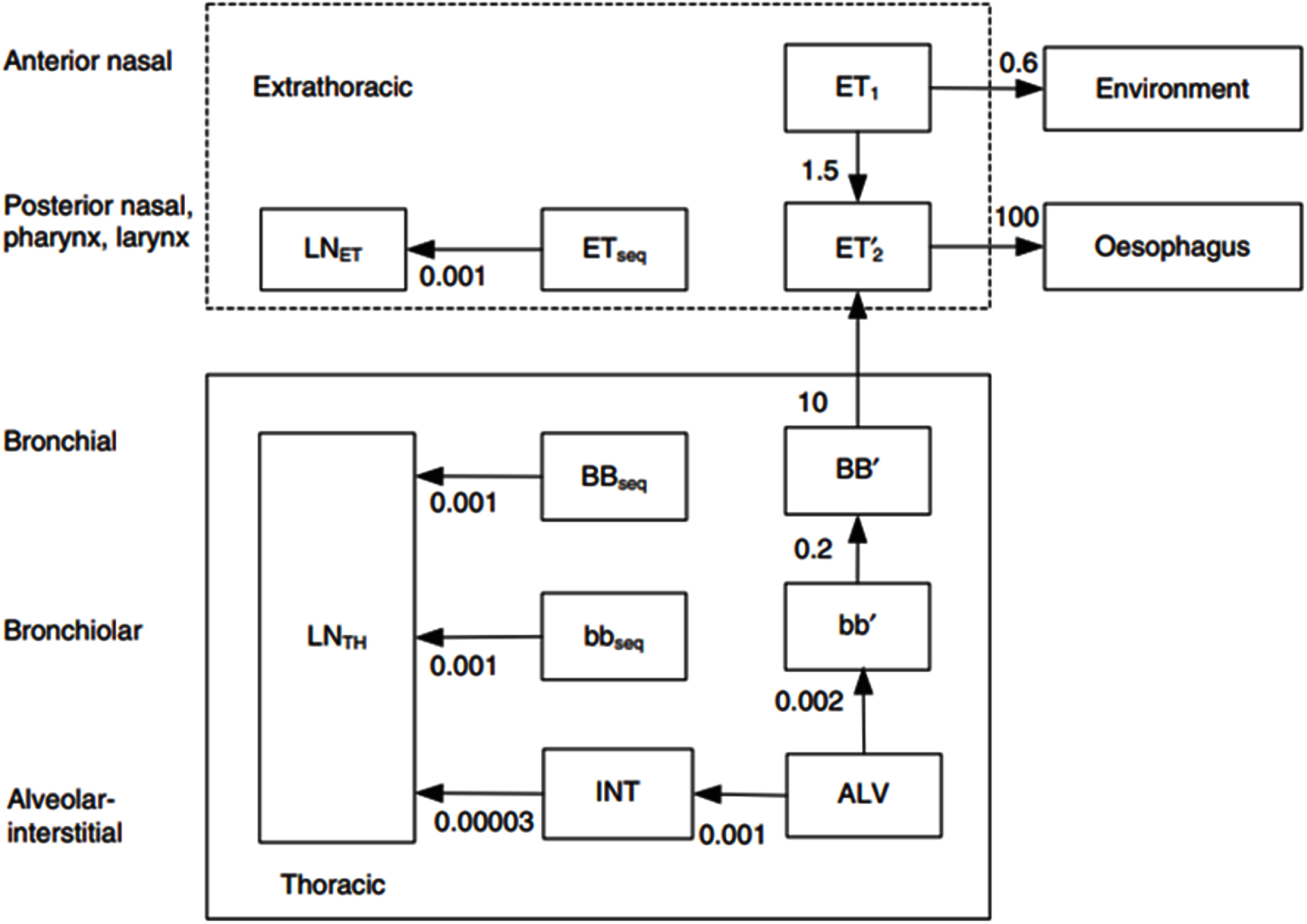
Compartment model of time-dependent particle transport from each respiratory tract regions of the revised HRTM. Arrows indicate the transfer of particles from one region to another with a designated transfer coefficient in units per day (d^−1^). *ET1*: anterior nose, *ETseq*: long-term retention in airway tissue of a small fraction of particles deposited in the nasal passages, *ET2prime*: short-term retention in *ET2, BBprime*: short term retention of particles in the Bronchial (*BB*), *bbprime*: short term retention of the particles in the Bronchiolar (*bb*), *BBseq*: long-term retention in airway walls in *BB* region, *bbseq*: long-term retention in airway walls in bb region, *LNET:* lymphatics and lymph nodes that drain the Extrathoracic (*ET*), *LNTH*: lymphatics and lymph nodes in the Thoracic (*TH*) region, *ALV*: retention of particles deposited in the alveoli, *INT*: very long-term retention of the particles deposited in the alveoli that penetrate to the interstitium. The HRTM was reproduced with permission from the ICRP Publication 130, figure 3.4 (ICRP 2015).

In the scope of this study, the Python programming language was adopted for the development of the biokinetic and dosimetry toolkit for subsequent stochastic analysis. The deterministic inhalation DC was reconstructed, following the recommendations of the ICRP (ICRP 2007, 2015). Figure 1 illustrates the methodological approach adopted for the computation and implemented in REDCAL for deterministic DC estimation, where 50 years was employed in this study as the committed period applicable for occupational intake of radionuclide. Biokinetic models represent the time-dependent material concentration incorporated in the body; the material could be a radioactive material, toxic material, or a drug. The complex mathematical framework resulting from the models can be solved numerically or algebraically. A comprehensive mathematical evaluation of a biokinetic model has been detailed elsewhere (Mate-Kole *et al* 2023, Mate-Kole and Dewji 2024). The ODEs posed by the inhaled ^131^I scenario were evaluated using the algebraic method utilized in the study by Mate-Kole *et al* (2023). Figure 2 depicts the coupled inhalation compartmental model for ^131^I as described in the ICRP Publication 130 and Publication 137 (ICRP 2015, 2017). The ICRP Publication 130 HRTM has been depicted as a single compartment in figure 2 for simplicity. However, it should be noted that the ICRP Publication 130 HRTM comprises a set of components representing the mechanical clearance of deposited radionuclides in the respiratory tract (figure 3).

### 2.2. Uncertain Parameters

Investigation of uncertain parameters was conducted through literature review of parameters that could potentially influence the HRTM and the biokinetic models. Previous studies have examined the sensitivity of lung dose to PD and clearance parameters using LUDUC (Bolch *et al* 2001, 2003, Huston *et al* 2003, Farfán *et al* 2004), based on the ICRP Publication 66 HRTM (ICRP 1994). The LUDUC study assessed the impact of parameter uncertainties in the deposition and clearance of inhaled particles (ranging from 0.001 *µ*m to 50 *µ*m) on lung doses from ^239^PuO_2_ and ^238^UO_2_/^238^U_3_O_8_. Our study leveraged these earlier studies to compile a list of uncertain parameters in the human respiratory tract. Through critical analysis of the literature, the volume of uncertain parameters was narrowed to 18 parameters (table 1). This selection incorporates the advancements reflected in the ICRP Publication 130 HRTM, highlighting progress made in the two decades between the ICRP Publication 66 and Publication 130 (ICRP 1994, 2015).

**Table 1.** Uncertain parameters utilized in the stochastic analysis.

Input	Description	Unit	Distribution	Mean/GM	SD/GSD
*Fn*	Fraction of air inhaled (Puncher 2014)	Unitless	Triangular	Mode = 1	Min: 0.4 Max: 1
*C_ae_(ET_1_)*	Random variable for aerodynamic deposition efficiency in *ET1* (Bolch *et al* 2001)	Unitless	Lognormal	1.00	1.82
*C_ae_(ET_2_)*	Random variable for aerodynamic deposition efficiency in *ET2* (Bolch *et al* 2001)	Unitless	Lognormal	1.00	1.82
*C_ae_(BB)*	Random variable for aerodynamic deposition efficiency in *BB* (Bolch *et al* 2001)	Unitless	Lognormal	1.00	1.58
*C_ae_(bb)*	Random variable for aerodynamic deposition efficiency in *bb* (Bolch *et al* 2001)	Unitless	Lognormal	1.00	1.58
*C_ae_(AI)*	Random variable for aerodynamic deposition efficiency in *AI* (Bolch *et al* 2001)	Unitless	Lognormal	1.00	1.3
*C_th_(ET_1_)*	Random variable for thermodynamic deposition efficiency in *ET1* (Bolch *et al* 2001)	Unitless	Lognormal	1.00	1.18
*C_th_(ET_2_)*	Random variable for thermodynamic deposition efficiency in *ET2* (Bolch *et al* 2001)	Unitless	Lognormal	1.00	1.18
*C_th_(BB)*	Random variable for thermodynamic deposition efficiency in *BB* (Bolch *et al* 2001)	Unitless	Lognormal	1.00	1.23
*C_th_(bb)*	Random variable for thermodynamic deposition efficiency in *bb* (Bolch *et al* 2001)	Unitless	Lognormal	1.00	1.23
*C_th_(AI)*	Random variable for thermodynamic deposition efficiency in *AI* (Bolch *et al* 2001)	Unitless	Lognormal	1.00	1.23
*f_d_(ET_seq_)*	Fraction of deposit in *ETseq* compartment (Bolch *et al* 2003)	Unitless	Lognormal	0.002	1.73
*f_d_(BB_seq_)*	Fraction of deposit in *BBseq* compartment (Bolch *et al* 2003)	Unitless	Lognormal	0.002	1.73
*f_d_(bb_seq_)*	Fraction of deposit in *bbseq* compartment (Bolch *et al* 2003)	Unitless	Lognormal	0.002	1.73
*L_ALV,INT*	Fractional clearance rate constant for mechanical clearance from *ALV* to *INT* (Puncher and Burt 2013)	d^−1^	Lognormal	median = 0.001	4.5
*L_ALV,bb*	Fractional clearance rate constant for mechanical clearance from *ALV* to *bb_prime* (Puncher and Burt 2013)	d^−1^	Lognormal	median = 0.002	3.2
*L_INT,LNTH*	Fractional clearance rate constant for mechanical clearance from *INT* to *LNTH* (Puncher and Burt 2013)	d^−1^	Lognormal	median = 0.00003	3
*Kpt*	Randomly sampled factor (Puncher and Burt 2013) from the given distribution to scale the following rates (*ET1* $ \to $ *ET2; ET1* $ \to $ *Env; ETseq* $ \to $ *LNET; ET2* $ \to $ *Oeso; BBseq* $ \to $ *LNTH; BB* $ \to $ *ET2; bbseq* $ \to $ *LNTH; bb* $ \to $ *BB*)	Unitless	Lognormal	Median of unity: 1	1.73

*Note: Oeso*—Oesophagus, *Env*—Environment compartment. *ET1*: anterior nose, *ETseq*: long-term retention in airway tissue of a small fraction of particles deposited in the nasal passages, *ET2*: Extrathroacic posterior nasal region, *BB*: Bronchial region, *bb*: Bronchiolar region, *BBseq*: long-term retention in airway walls in *BB* region, *bbseq*: long-term retention in airway walls in bb region, *LNET*: lymphatics and lymph nodes that drain the Extrathoracic (*ET*), *LNTH*: lymphatics and lymph nodes in the Thoracic (*TH*) region, *ALV*: retention of particles deposited in the alveoli, *INT*: very long-term retention of the particles deposited in the alveoli that penetrate to the interstitium, The ‘prime’ indicates short terms retention in a compartment. It is important to note that the LHS algorithm implemented accepts GM and GSD as inputs for the lognormal distribution parameters. Therefore, all lognormal distribution bounds in this table were converted to GM and GSD.

The comprehensive list of uncertain parameters under consideration, which includes statistical bounds and confidence intervals, such as mean or geometric mean (GM), standard deviation (SD) or geometric standard deviation (GSD), minimum, maximum, and mode depending on the distribution, was then input into an in-house computational module for LHS.

Specifically, the ICRP (ICRP 1994) provides mathematical expressions to evaluate both aerodynamic (inertial impaction and gravitational settling) and thermodynamic (particle diffusion) deposition efficiencies in five regions of the HRTM (*ET1, ET2, BB, bb, AI*). The deposition efficiencies are defined in terms of *a, R*, and *p* as demonstrated in ICRP Publication 66 (ICRP 1994). These mathematical expressions are derived from regression analyses of experimental data and sophisticated theoretical models for the extrathoracic and thoracic regions, respectively. To propagate uncertainty, the ICRP recommends introducing a random variable multiplier, *C*, for each region and for both the aerodynamic and thermodynamic components (Bolch *et al* 2001). The random variables, *C*, are assigned lognormal distributions with GMs of one and GSDs given as $\sqrt {{c_i}} $, where ${c_i}$ represents scaling constants proposed by ICRP Publication 66 (ICRP 1994), Table 14, to estimate the upper or lower 95% confidence bound of the regional depositions (Bolch *et al* 2001). The random variables are then used to multiply the fitting parameters, ${a_i}$, in the deposition efficiency expressions. Similar to this study, the mathematical expressions proposed by ICRP Publication 66 were implemented to compute the regional PD efficiencies. Consequently, the random variable *C* for each region of the HRTM and for each component (aerodynamic and thermodynamic) was adopted. It is worth noting that since 1995, the approach of assigning lognormal distributions to random variable multipliers has been consistently used due to a lack of new data and the difficulties in validation. For example, even though the ICRP has recommended an updated HRTM (ICRP 2015), no changes were made to the ICRP human respiratory tract PD model, except for the redistribution of deposits in the extrathoracic region. The same baseline deposition model was utilized in this study.

For deposited material in each region of the HRTM, it is assumed that a portion of the deposit is sequestered (long-term retention in airway tissue) in *ETseq, BBseq,* and *bbseq* (ICRP 1994, 2015). The fraction (*f_d_*) of the deposit in the sequestered compartments is considered uncertain in this study, as introduced by Bolch *et al* (2003), and was assigned a lognormal distribution. The GM adopted in this study was the updated reference fraction (0.002) with a GSD based on an uncertainty factor (UF) of 3 for a 95% confidence interval (Bolch *et al* 2003).

Additionally, the distribution of the fraction of air inhaled (*Fn*) utilized in this study was based on measurements of inter-subject variation in nose-breathing adults (Puncher 2014). The *Fn* parameter was assumed to follow a triangular distribution with a minimum value of 0.4 and a maximum value of 1. Moreover, Makumbi *et al* (2024) reviewed parameter uncertainty analysis of equivalent lung dose for miners, referencing the *Fn* distribution from Marsh and Birchall (2009), which had a minimum of 0.3 and a maximum of 1. However, for this study, the minimum value of *Fn* was chosen to be 0.4 to reflect inter-subject variation and to adjust the right-angled triangular distribution to align with the minimum *Fn* proposed for a light worker (sitting activity and light exercise as defined by ICRP Publication 66 (ICRP 1994) and adopted in ICRP Publication 130 (ICRP 2015). The HRTM assumes an *Fn* value of 1 for a nose augmenter engaged in sitting activity and light exercise, and an *Fn* value of 0.7 for sitting and 0.4 for light exercise for a mouth breather.

Having discussed the selected uncertain parameters influencing PD, it is essential to transition to the next critical component of the inhalation-biokinetic model included in the stochastic analysis: mechanical clearance, or particle transport. Particle transport depicts the movement of particles towards the alimentary tract and lymph nodes (ICRP 2015). According to Puncher and Burt (2013), the movement of material from the alveolar-interstitial (*AI*) region to the bronchiolar region and thoracic lymph nodes is facilitated by macrophages. Consequently, the distributions of the particle transport rates from the AI region (primarily from *ALV* to *bb, ALV* to *INT*, and *INT* to *LNTH*) are based on data fitted by Gregoratto *et al* (2010). It is important to note that, although Puncher and Burt (2013) used 0.0013 as the median particle transfer rate from *ALV* to *bb*, this study selected 0.002 as the median - the reference particle transfer rate, based on ICRP Publication 130. The remaining particle transport rates, assumed to be correlated, were scaled by a factor *Kpt*, which follows a lognormal distribution with a median of 1 and a GSD of 1.73 (Puncher 2014). These distributions were adopted in this study, and the factor *Kpt* is refered to as *K_factor* in the analysis.

### 2.3. Stochastic expansion

The stochastic expansion subsection details the computational implementation of the LHS method, the propagation of uncertainty and sensitivity analysis, and the characterization of distribution of the inhalation committed effective dose coefficient (CEDC). LHS is a stratified sampling technique in which the range of each uncertain parameter is divided into strata of equal probability (Dalbey *et al* 2021). LHS requires fewer samples than traditional Monte Carlo for the same accuracy in statistics (McKay and Beckman 2000). A sample size of 10 000 was utilized for the LHS sampling approach to achieve increased resolution and statistical power. A study conducted by Stein (Stein 1987, McKay 1992) demonstrated that the LHS method yields an asymptotically smaller variance as compared to the standard random sampling approach. The criterion for the sample size selection was analyzed through sample size calculations. The 18 uncertain parameters were randomly sampled according to their respective distributions. The sample size was then determined using the SD of the summary statistics and a desired precision level of 0.000 091. The calculated sample size was predicted to be 9990, which was rounded up to 10 000. The uncertain parameters were input into an in-house Python LHS computational module, and the sampling was performed using *pyDOE2* (Sjögren and Svensson 2018).

Moreover, in inhalation scenarios, the ICRP provides PD values in the respiratory tract in tabulated form. However, to accurately propagate uncertainty through the HRTM and enable flexibility, the ICRP deposition model was explicitly coded into REDCAL following the ICRP PD methodology (ICRP 1994). The fraction of inhaled aerosol in the respiratory tract region is referred to as deposition efficiency. As outlined in ICRP Publication 66 (ICRP 1994), the deposition efficiency (DE) (equation (1)) for inhalation and exhalation is computed using empirical relations of filtration efficiencies and volumetric factors of each region of the human respiratory tract,

\begin{equation*}{\text{D}}{{\text{E}}_j} = {\text{ D}}{{\text{E}}_{j - 1}}{\eta _j}\frac{{{\phi _j}}}{{{\phi _{j - 1}}}}\left( {\frac{1}{{{\eta _{j - 1}}}} - 1} \right){\text{ for }}j = 1,N{ }\end{equation*}

where: ${\eta _j}$ is filtration efficiency of region *j*—fraction of aerosol entering the region that is deposited in that region and ${\phi _j}$ is the volumetric factor of region *j*—fraction of initially inhaled air that reaches that region. The deposition fractions obtained with the in-house deposition computational module were compared with the ICRP published deposition values. It is important to note that a reference worker (i.e. the subject of the case explored in this study) is assumed to engage in mixed respiratory activities, predominantly resting and light exercise. As such, the deposition computational module was customized to compute the deposition efficiencies for the pure activities and then weighted the average by the fraction of air breathed during each activity. An in-depth analysis of the computational methodology of the ICRP PD model is detailed in the ICRP Publication 66 and the KDEP study by Klumpp and Bertelli (ICRP 1994, Klumpp and Bertelli 2017).

The 10 000 observations of the 18 uncertain parameters were then integrated into REDCAL for a global sensitivity analysis, where all the uncertain parameters were allowed to vary over their full range simultaneously. Thus, REDCAL computed 10 000 sets of vectors of ^131^I type F inhalation CEDC following the methodology for occupational intake of radionuclides.

Additionally, to rank the most impactful parameters, a RF regression model coupled with SHAP was employed to improve transparency and understanding of the machine learning model. Classically, correlation and regression coefficient approach can be used to quantify the most impactful uncertain parameters. For multiple parameters, a multiple linear regression model was deemed an appropriate model approximation for sensitivity analysis, assuming the model is linear (Huston 1995, Bolch *et al* 2001). A study by Bolch *et al* (2001) employed techniques proposed by the International Atomic Energy Agency (IAEA) safety series (IAEA 1989) for model reliability assessment. The LUDUC study (Huston 1995) utilized the coefficients of the multiple linear regression formalism based on rank-transformed data set, such as the standardized rank regression coefficients (SRRC). The SRRC provides a standardized measure of variable importance by ranking independent variables (Iman *et al* 1985). Each variable’s smallest value is assigned a rank of 1, with subsequent values incremented by one unit up to the largest value (Iman *et al* 1985). The standardization is achieved by transforming variables based on the mean $\bar X$ and SD ${\sigma _X}$, calculated as ${X^*} = \left( {X - \bar X} \right)/{\sigma _X}$, effectively eliminating unit mismatches in the multivariate model (Iman *et al* 1985).

Notwithstanding, the approach employed in this study instead hypothesizes that the complexities of model parameter interactions can be underestimated with classical linear regression. While SRRC addresses non-linearity through rank transformation, the model still assumes linearity after the transformation. To address this, our study harnessed a machine learning RF regression model to provide a comprehensive view of the analysis and enhance feature importance classification.

The RF regression model measures the decrease in variance reduction that a feature brings when used independently in a decision tree. Essentially, this tree-based model was investigated for its ability to outperform standard deep learning models on tabular-style datasets (Lundberg *et al* 2020). Furthermore, SHAP stands out due to several advantages (Hamilton and Papadopoulos 2023), including flexibility in providing local-to-global explanations, enhancing interpretability without sacrificing accuracy, and an architecture derived from the theoretical foundation of cooperative game theory.

To perform the RF regression analysis, the CEDC—used as the target response metric for this study—were normalized via min–max scaling. This transformation adjusted the widely varying ranges of the response dataset to a fixed range (0–1), ensuring that the RF effectively captured the relationships in the data. A test size of 25% was allocated, and a grid search was conducted to evaluate the optimal number of trees to use in the RF regression model. Using the *RandomForestRegressor* from scikit-learn in Python (Pedregosa *et al* 2011), the regression analysis was conducted for feature importance based on Gini impurity-based feature importances, where the importance of the feature is estimated based on how much each feature contributes to reducing the impurity across all the decision trees. The maximum depth of the RF regression model was set to default which ensures that the nodes are expanded until all leaves were pure. Model performance was evaluated by computing the root mean squared error (RMSE) and the mean absolute error (MAE) using scikit-learn (Pedregosa *et al* 2011). The RMSE measures the average difference between the values predicted by the model and the actual values, while the MAE measures the average absolute difference between the predicted values and the actual response values. The RF regression model was incorporated into a SHAP explainer for comprehensive model interpretation.

To characterize the distribution of the CEDC, a non-parametric goodness of fit (K–S) test was conducted. The K–S test operates under the assumption of a null hypothesis that the two samples are drawn from the same distribution (Massey 1951, Berger and Zhou 2014, Virtanen *et al* 2020). The test rejects this null hypothesis if there is evidence to suggest that the two samples come from different distributions (Virtanen *et al* 2020). For this study, the Python SciPy *kstest* module (Virtanen *et al* 2020) was employed for a K–S test to compare the underlying distribution of the CEDC against a given theoretical distribution. The theoretical distributions considered were the continuous distributions available in the *scipy.stats* module (Virtanen *et al* 2020). The statistical parameters such as the minimum, maximum, mean, SD, and percentiles (2.5th, 25th, 50th, 75th and 97.5th) were also inferred. Consequently, uncertainty is expressed in terms of lower (2.5th percentile—Q_L_) and upper (97.5th percentile—Q_u_) bounds, *X* and *Y*, such that there is approximately a 95% probability that the true central value lies between *X* and *Y*. The central estimate, *C*, is used to determine the UF, which is calculated as the maximum of *C*/*X* and *Y*/*C* (Leggett 2001). This approach allows for expressing the range of uncertainty around a central estimate.

## 3. Results

To facilitate a flexible uncertainty and sensitivity analyses, the complexities of the ICRP PD model were decoded and reconstructed into a custom in-house Python PD module, which was subsequently integrated into REDCAL as REDCAL_dep_. The computational module underwent benchmarking against the ICRP-published deposition fractions derived from the ICRP Publication 130 HRTM. This benchmarking covered AMADs ranging from 0.3 *µ*m to 20 *μ*m, with relative and absolute differences reported as given in the data in table 2.

**Table 2.** The percent relative difference (RD %) and absolute difference (AD %) in computing the PD in the human respiratory tract between the calculated values and the ICRP published deposition fractions (ICRP Publication 130 (ICRP 2015) for a reference worker.

AMAD (*µ*m)	Total deposition (RD *%*)	Total deposition (AD *%*)
0.3	0.67	0.20
0.5	0.00	0.00
0.7	−0.24	−0.10
1	−0.20	−0.10
2	−0.29	−0.20
3	−0.38	−0.30
5	−0.61	−0.50
7	−0.62	−0.50
10	−0.78	−0.60
15	−0.84	−0.60
20	−1.04	−0.70

Note: *AMAD*—Activity Median Aerodynamic Diameter.

**Figure 4.**
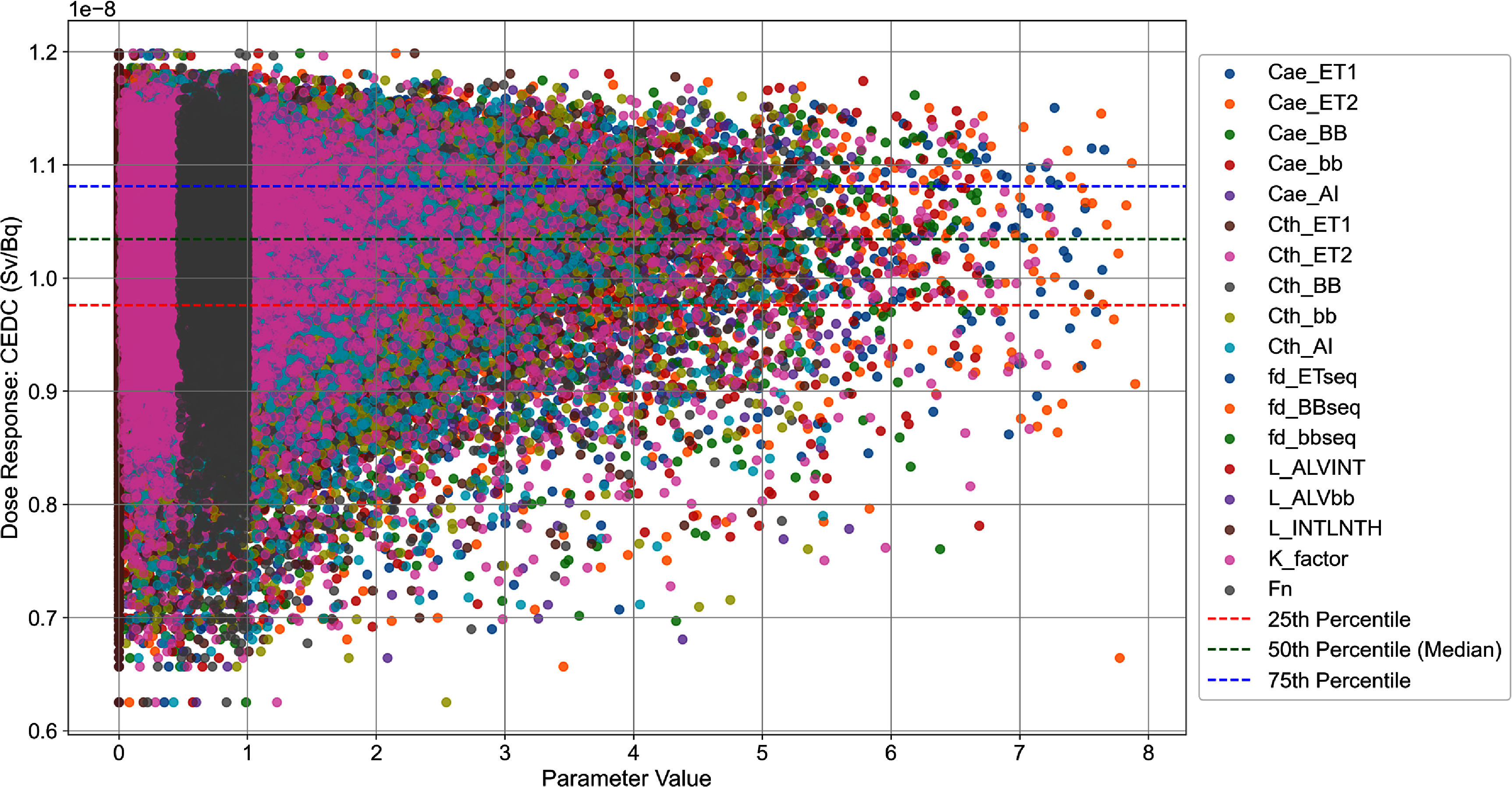
Percentile plot illustrating the distribution of the committed effective dose coefficients (CEDC) as a function of the uncertain parameter sample points for the 10 000 sample observations. Each dot, represented by a distinct color, corresponds to the sample value of an uncertain parameter obtained using latin hypercube sampling (LHS) and its resulting CEDC. The dotted lines represent the percentiles of the underlying distribution. The parameter value indicated in the plot depicts the sampled value of each uncertain parameter considered as listed in table 1.

The summary statistics for the CEDC for inhaled ^131^I type F, with a lognormally distributed particle size of 5 *µ*m, were estimated as follows: mean = $1.02 \times {10^{ - 8}}{\text{ Sv}}/{\text{Bq}}$, SD = $8.10 \times {10^{ - 10}}{\text{ Sv}}/{\text{Bq}}$, minimum = $6.25 \times {10^{ - 9}}{\text{ Sv}}/{\text{Bq}}$, 2.5th percentile $= 8.31 \times {10^{ - 9}}{\text{ Sv}}/{\text{Bq}}$, 25th percentile = $9.76 \times {10^{ - 9}}{\text{ Sv}}/{\text{Bq}}$, 50th percentile = $1.03 \times {10^{ - 8}}{\text{ Sv}}/{\text{Bq}}$, 75th percentile = $1.08 \times {10^{ - 8}}{\text{ Sv}}/{\text{Bq}}$, 97.5th percentile $ = 1.14 \times {10^{ - 8}}{\text{ Sv}}/{\text{Bq}}$, and maximum = $1.20 \times {10^{ - 8}}{\text{ Sv}}/{\text{Bq}}$. Figure 4 illustrates the percentile plot for the DC, demonstrating the distribution of the sample points of the 18 uncertain parameters. It is important to emphasize that the deterministic CEDC estimated with REDCAL for the exposure scenario described in this study was $1.11 \times {10^{ - 8}}{\text{ Sv}}/{\text{Bq}}$—a value that is consistent with the ICRP published occupational intake of radionuclide CEDC $\left( {1.1 \times {{10}^{ - 8}}{\text{ Sv}}/{\text{Bq}}} \right){ }$(ICRP 2017).

**Figure 5.**
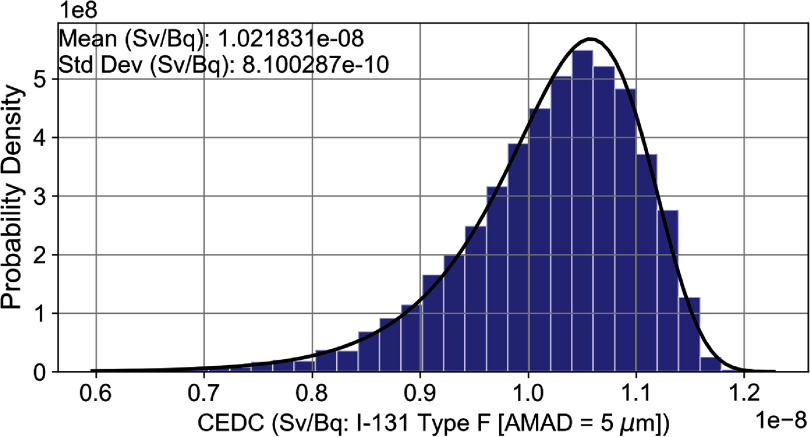
Best fit distribution (log-gamma) of the committed effective dose coefficient (CEDC) for inhaled ^131^I type F. Std Dev indicates the standard deviation of the CEDC samples.

**Figure 6.**
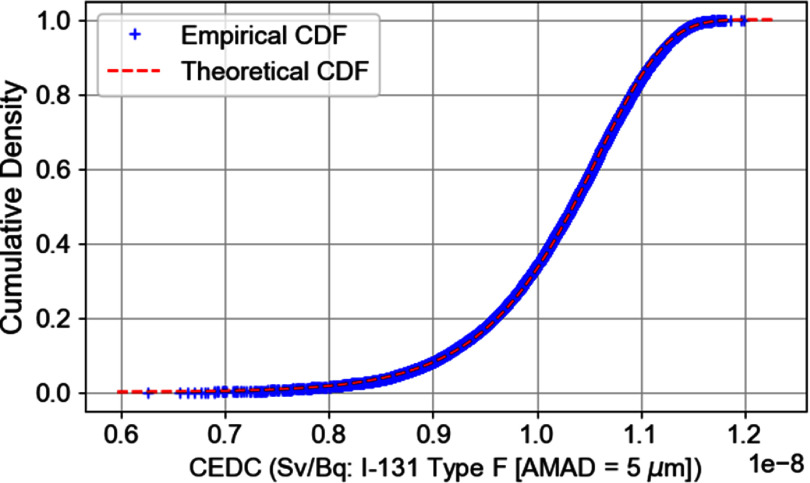
Cumulative density function (CDF) plot comparing the underlying distribution of the committed effective dose coefficient (CEDC) and the predicted best fit distribution.

A nonparametric K–S test was performed using a one-sample, two-tailed approach. The test calculated the difference (denoted as *D*_max|eCDF-tCDF|_) between the empirical cumulative distribution function (eCDF) of the CEDC and the specified theoretical distributions (tCDF). The optimal distribution is determined as the one with the smallest maximum difference. For this study, the best fit distribution predicted was log-gamma distribution (figure 5) with a *D*_max|eCDF-tCDF|_ value of 0.011 and a *p*-value of 0.2. Further investigation was conducted based on the cumulative density functions for a better presentation of the deviations, if any. Figure 6 illustrates the cumulative density plot comparing the underlying distribution of the CEDC and the best fit distribution for all the 10 000 observations.

**Figure 7.**
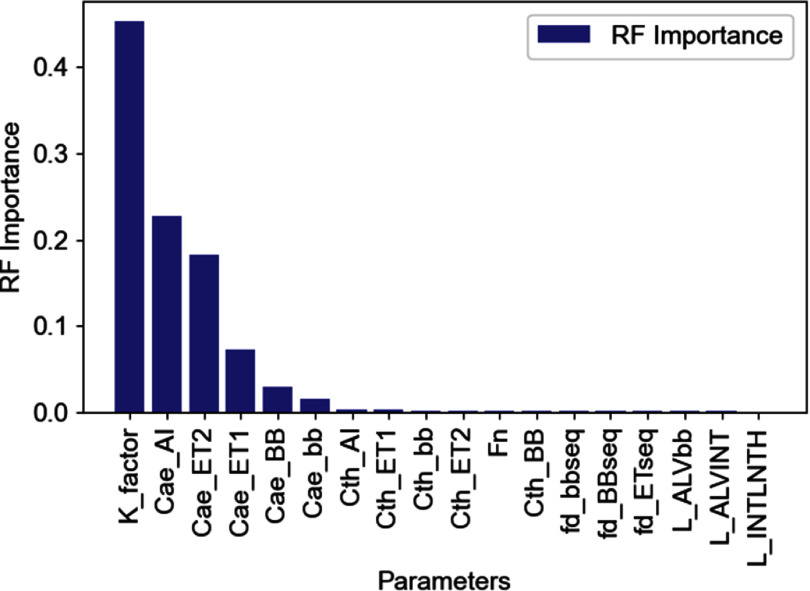
Parameter importance of uncertain parameters with the committed effective dose coefficient (CEDC) as the target response. *Note:* the descriptions of the parameters have been provided in table 1 and *K_factor* corresponds to *Kpt*.

**Figure 8.**
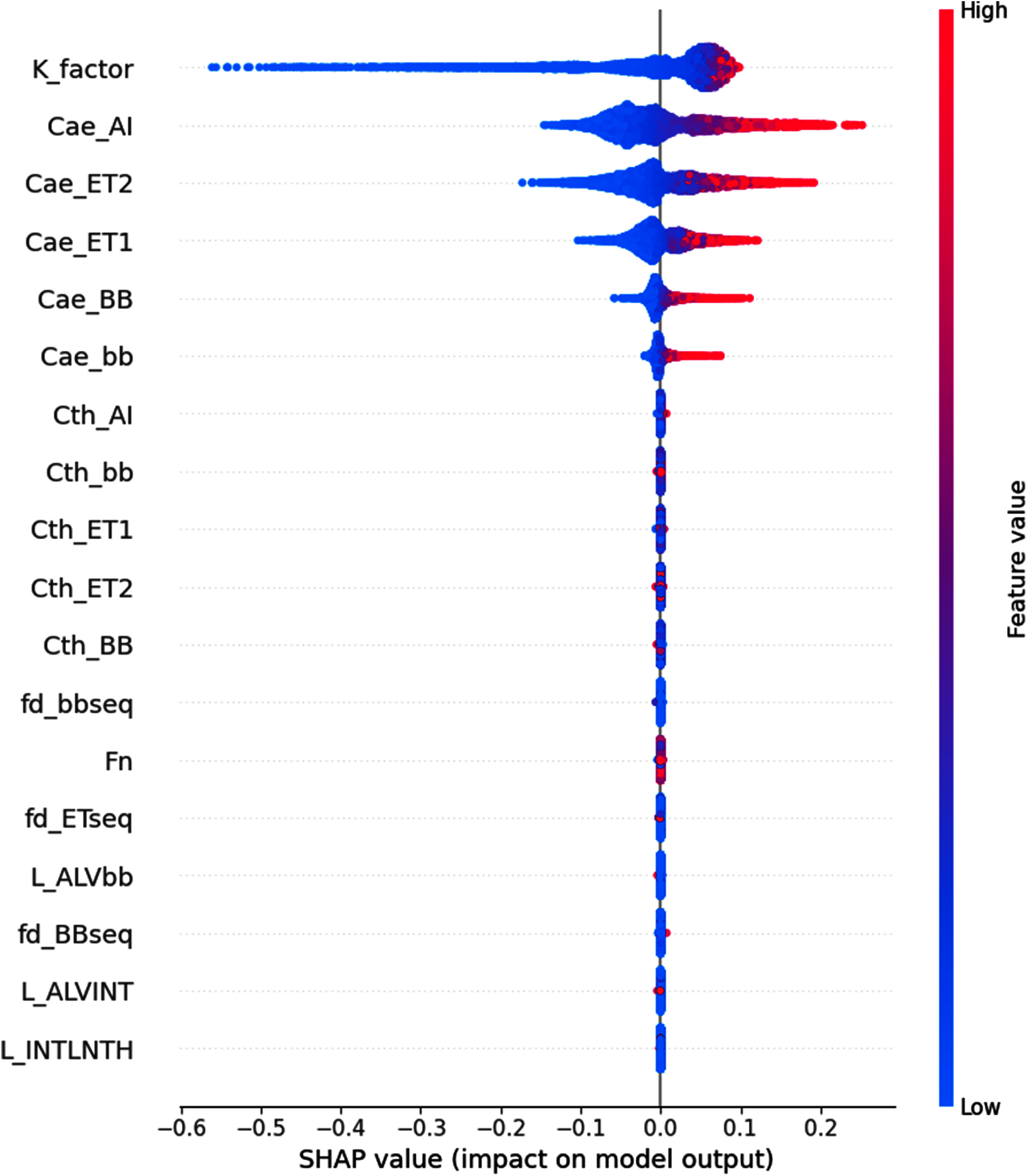
Parameter importance of uncertain parameters with the committed effective dose coefficient (CEDC) as the target response integrating SHAP for extended explanation. Note: the descriptions of the parameters have been provided in table 1 and *K_factor* corresponds to *Kpt*.

The most impactful uncertain parameters in the inhalation-biokinetic model for iodine were identified utilizing RF regressor integrated with SHAP. A grid search determined that 150 estimators were optimal determining the number of trees in the forest. Figure 7 depicts the ranking of most impactful parameters for the inhalation scenario. The *x*-axis of figure 7 indicates the uncertain parameters considered, while the *y*-axis shows the feature importance predicted by the RF model (RF importance). Additionally, figure 8 provides an expanded explanatory plot, offering a detailed analysis of each parameter’s impact on the dependent variable (CEDC) using SHAP. The obtained MAE and RMSE for the training data were 0.007 and 0.009, respectively. For the test data set, the obtained MAE and RMSE were 0.018 and 0.027, respectively. These results indicate that the model has a lower error on the training data compared to the test data, which is expected as the model is trained on this data. The differences between the training and test errors suggest that the model generalizes reasonably well to unseen data.

It is worth noting that although the CEDC is on the order of ${10^{ - 8}}$, where min–max scaling of the response fed into the RF regression model constrained the CEDC values to the range of 0–1, with the mean of the normalized response being 0.691 Sv/Bq.

The uncertainty in the CEDC was quantified using the UF. In this study, the UF was determined to represent an interval of true values with respect to the mean of the distribution of the CEDC, with a 95% confidence interval. The ICRP reference CEDC was used as the central estimate. The 2.5th percentile of the acquired distribution was taken as the lower bound (Q_L_), and the 97.5th percentile was taken as the upper bound (Q_U_). Table 3 shows the quantitative results of the UF and compares the instance of using all 18 uncertain parameters in the global sensitivity analysis with the instance of varying only the *K_factor* and *Cae_AI*.

**Table 3.** Uncertainty factors for the inhalation committed effective dose coefficient (CEDC) for ^131^I (type F) with a particle size of 5 *µ*m.

Quantity	All uncertain parameters	Only *K_factor*	Only *Cae_AI*
ICRP CEDC	$1.10 \times {10^{ - 8}}{\text{ Sv}}/{\text{Bq}}$	$1.10 \times {10^{ - 8}}{\text{ Sv}}/{\text{Bq}}$	$1.10 \times {10^{ - 8}}{\text{ Sv}}/{\text{Bq}}$
Q_L_	$8.45 \times {10^{ - 9}}{\text{ Sv}}/{\text{Bq}}$	$8.85 \times {10^{ - 9}}{\text{ Sv}}/{\text{Bq}}$	$1.07 \times {10^{ - 8}}{\text{ Sv}}/{\text{Bq}}$
Q_U_	$1.14 \times {10^{ - 8}}{\text{ Sv}}/{\text{Bq}}$	$1.11 \times {10^{ - 8}}{\text{ Sv}}/{\text{Bq}}$	$1.14 \times {10^{ - 8}}{\text{ Sv}}/{\text{Bq}}$
Q_U_/ICRP	1.04	1.01	1.04
ICRP/Q_L_	1.32	1.24	1.03

## 4. Discussion

The primary aim of the study was to conduct a stochastic analysis of uncertain biokinetic parameters in the human respiratory tract by performing uncertainty and sensitivity analyses of ^131^I of absorption type F inhalation scenarios. In occupational, as well as emergency scenarios following nuclear and radiological accidents, various needs may arise, including but not limited to accelerated breathing or respiratory activities, individuals with specialized health conditions, and sex- and age-specific metabolism and physiology. To address these needs, the current and up-to-date reference inhalation-biokinetic models were expanded to assess the uncertainty and variability associated with predefined deterministic parameters. To establish the computational framework for further stochastic analysis, the methodologies of ICRP DC, including the ICRP PD model, were reconstructed in the Python programming language.

The PD fractions computed for a reference worker, who is a nose breather, using the in-house deposition computational module, were compared to those of the ICRP published deposition fractions. The relative and absolute differences for the AMAD ranging from 0.3 *µ*m to 20 *µ*m for total deposition were found to be 1.04% and 0.7%, respectively (table 2). These represented the maximum differences in the total PD fractions obtained for both under- and over-estimation, indicating a good agreement with the published data. Furthermore, the estimated deterministic CEDC showed excellent agreement with the ICRP published inhalation DC, demonstrating the robustness of REDCAL.

The statistical analysis conducted in this study revealed that the point estimate of the CEDC provided by the ICRP utilizing the ICRP Publication 130 HRTM ($1.1 \times {10^{ - 8}}{\text{ Sv}}/{\text{Bq}}$—ICRP Publication 137 (ICRP 2017)) slightly exceeds the 75th percentile of the statistical distribution obtained in this study, particularly for the inhalation of ^131^I of type F. This suggests that for the uncertain parameters evaluated in this study, there exists a difference from the current deterministic DC models, as the published point estimate falls within the top 25% of the predicted distribution. The observed difference between the mean and median of the CEDC distribution in this study and the reference CEDC point estimate by the ICRP likely attributable to the following factors. Firstly, the uncertain parameters used in this study were specifically related to the human respiratory tract. The global sensitivity analysis was conducted by simultaneously considering all the uncertain parameters in table 1, while keeping all systemic and dissolution rates constant at their ICRP reference values. Additionally, uncertainties in the dosimetric (*S*-coefficient) model were not included in this study. These factors may contribute to the reference DC value lying slightly above the 75th percentile. Although the reference value lies slightly above the 75th percentile, it is still below the 97.5th percentile. Furthermore, the fraction of air inhaled distribution (*Fn*) used in the study was based on measurements of inter-subject variation in nose-breathing adults (Puncher 2014). The *Fn* parameter was assumed to follow a triangular distribution with a minimum value of 0.4 and a maximum value of 1. This parameter distribution could also contribute to the reference DC’s slight offset above the 75th percentile since the ICRP generated the reference point estimate assuming that air is inhaled predominantly through the nose with an *Fn* value of 1.0. These findings emphasize the importance of evaluating uncertainty and variability in dose estimation models to ensure their accuracy in real-world scenarios.

A nonparametric K–S test was conducted to infer the probability distribution function. In this case study, the best-fit predicted distribution was found to be the log-gamma distribution, with a maximum distance between the cumulative distributions of 0.011 and a corresponding *p*-value of 0.2. The K–S test was performed based on a two-sided hypothesis. According to the two-sided test, the null hypothesis is that the two distributions are identical. Thus, a *p*-value greater than a certain significance level (set to 5% in this study) provides no significant evidence to reject the null hypothesis. The *p*-value obtained for the log-gamma distribution was 20% , indicating that the theoretical distribution and the empirical distribution of the CEDC are identical. It is worth noting that the K–S statistic alone—the maximum distance between the cumulative distributions—is not informative enough for conclusive evidence of the best-fit distribution; hence, the importance of the *p*-value. It was informative to analyze the cumulative distribution functions of the best-fit theoretical distribution and the underlying distribution of the inhaled ^131^I CEDC. Figure 6 compared the underlying distribution of the inhalation CEDC and the best-fit theoretical distribution, for which the predictions show excellent agreement with the theoretical distribution. Understanding that the inhalation CEDC for the case study follows a log-gamma distribution allows for a reliable representation of the anatomical and physiological characteristics of the human respiratory tract, based on the ICRP Publication 130 HRTM. This enhances the precision of real-world dose predictions, informing protective action decisions in the event of the release of radioactive ^131^I. Additionally, the probabilistic representation of the inhalation DC improves the reliability of the DC by providing insight into the statistical bounds.

Furthermore, incorporating a RF regression model coupled with SHAP revealed that altering the AI regional value of the respiratory tract significantly affects the overall inhalation DC value for this case study, in the absence of parameters resulting in compounding uncertainties. Specifically, figures 7 and 8 illustrate the ranking of the most impactful uncertain parameters, with the CEDC as the dependent variable. Essentially, it was predicted that variations in *K_factor* (HRTM particle transport rates scaling factor) and *CaeAI* (random variable for aerodynamic deposition efficiency in the AI region), have a significant impact on the predicted CEDC. This prediction is consistent with the inherent complexities involved in modeling the human respiratory tract. Thus, in the context of this study, *K_factor* has the strongest relationship with the inhalation CEDC for the inhaled ^131^I scenario, followed by *CaeAI*, explaining the greatest amount of the variance. Although *K_factor* was identified as the most important uncertain parameter, it scales rates that are assumed to be correlated. Therefore, small changes in *K_factor* can lead to significant variations in model predictions due to compounding uncertainty. Additionally, the ICRP utilizes empirical relations directly based on experimental data to estimate the PD in the human respiratory tract for eventual dose estimation. The *CaeAI* parameter is a random variable used as a multiplier of a fitting parameter (*a*) in the ICRP deposition model, mainly in the alveolar-interstitial region for aerodynamic deposition efficiency. It is important to note that the fitting parameter (*a*) and the other parameters defining the expressions of the respiratory tract deposition model were estimated based on regression analyses of deposition data leveraging from sophisticated theoretical models in literature (Huston 1995).

Moreover, figure 8 depicts a multi-directional interpretation of the impact of the uncertain parameters. The SHAP approach also ranked *K_factor* and *CaeAI* as the most impactful (figure 8). Particularly, instances of high feature values (red) extending towards the positive SHAP value region indicate an increase in the predicted CEDC, while extending towards negative SHAP values depicts a decrease in the CEDC. On the other hand, lower feature values (blue) extending towards positive SHAP values signify an increase in the CEDC, and extension towards negative SHAP values depicts a decrease in the CEDC.

In the context of the highly ranked most impactful uncertain parameter, *K_factor*, it was observed that lower values contribute predominantly to a reduction in the inhalation CEDC. Regarding the second most impactful uncertain parameter, *CaeAI*, it was observed that a cluster of the feature values is low, contributing to the reduction in the CEDC. However, the impact of the higher feature values on increasing the DC is higher relative to the tendency for a decrease in the DC. Nonetheless, variations in this parameter, which constitute variations in the fitting parameter in the AI region, significantly impact the inhalation CEDC.

The findings of this study are consistent with those of Huston *et al* (2003), who attributed 50% of the total lung dose variability to the dose to the AI region, associating 90% of the variability in the dose to the AI region with uncertainties in the ventilation rate (the quantity of air inhaled or exhaled per unit time), the AI deposition fraction, and specific clearance rates in the compartmental model based on the ICRP Publication 66 HRTM (ICRP 1994), though solely for ^239^PuO_2_. This study incorporated the updated ICRP respiratory tract model (ICRP Publication 130 HRTM (ICRP 2015)), integrating iodine systemic model for assessing the distribution and CEDC for inhaled ^131^I. Similarly, this study revealed that changes in the AI region significantly affect the DC and, consequently, the dose due to radiation exposure via inhalation, attributing approximately 25% of the impact to the *CaeAI* uncertain parameter. Furthermore, the UF obtained was 1.32 (max | ICRP/Q_L_, Q_U_/ICRP |), taking into account all the 18 uncertain parameters for the sensitivity analysis (table 3). However, considering only *K_factor* and *Cae_AI* resulted in UF of 1.24 and 1.04, respectively. Quantitatively, 93.90% and 78.59% of the uncertainty in the CEDC was predicted to be due solely to *K_factor* and *Cae_AI,* respectively, based on this study—an observation that validates the prediction of the machine learning model.

The integration of the machine learning technique into the framework provided a comprehensive approach for the stochastic analysis for feature importance classification. Essentially, this is the first analysis of its kind of uncertain parameters in the HTRM to incorporate a machine learning model for uncertainty and sensitivity analysis. The results therefore provide insight into directing future research efforts in respiratory tract modeling ensuring stakeholders make the important changes when updating the models. In addition, the predicted distribution depicts the variability and uncertainty in the CEDC associated with the inhaled ^131^I of type F, thus providing a realistic representation of the real-world complexities compared to the point estimates.

## 5. Conclusions

Radiation and medical countermeasure applications, as well as dose reconstruction in radiation epidemiology cohort studies from nuclear and radiological exposures, require careful consideration of variability and uncertainties in biological responses. The aim of this study was to assess the variability in deterministic biokinetic and dosimetry models and to better represent a stochastic consideration of radionuclide metabolism resulting from realistic radionuclide source term intakes. Specifically, stochastic analysis of uncertain biokinetic parameters in the human respiratory tract was conducted through uncertainty and sensitivity analysis of ^131^I of absorption type F, incorporating the ICRP Publication 130 HRTM for inhalation DC estimation. The adoption of nonparametric K–S tests and machine learning techniques, such as RF and SHAP, has enhanced the statistical interpretation of the stochastic effects in the biokinetic and dosimetric model beyond point estimates, revealing significant impacts of uncertain parameters. Notably *K_factor* and *CaeAI*, identified as the most impactful parameters, significantly influence the predicted inhalation DC. This study provides a comprehensive stochastic framework for evaluating uncertainties in biokinetic and dosimetric models, offering enhanced insight into the variability of inhalation DCs, particularly for radionuclide exposure scenarios, and improving the accuracy of risk assessment and medical countermeasure development.

## Data Availability

All data that support the findings of this study are included within the article (and any supplementary files).

## References

[jrpad7ec3bib1] Berger V W, Zhou Y (2014). Wiley statsref: Statistics reference online.

[jrpad7ec3bib2] Bertelli L, Lipsztein J L, Melo D R, Puerta A, Wrenn M E, Dorado-Carrera A E (1997). Biokinetic models for the metabolism of uranium: an overview.

[jrpad7ec3bib3] Bolch W E, Farfán E B, Huh C, Huston T E (2001). Influences of parameter uncertainties within the ICRP 66 respiratory tract model: particle deposition. Health Phys..

[jrpad7ec3bib4] Bolch W E, Huston T E, Farfán E B, Vernetson W G, Bolch W E (2003). Influences of parameter uncertainties within the ICRP-66 respiratory tract model: particle clearance. Health Phys..

[jrpad7ec3bib5] Chen S Y, Tenforde T (2010). Optimization approaches to decision making on long-term cleanup and site restoration following a nuclear or radiological terrorism incident. Homel. Secur. Aff..

[jrpad7ec3bib6] Dalbey K, Eldred M, Geraci G, Jakeman J, Maupin K, Monschke J A, Seidl D, Tran A, Menhorn F, Zeng X (2021). Dakota A Multilevel Parallel Object-Oriented Framework for Design Optimization Parameter Estimation Uncertainty Quantification and Sensitivity Analysis: Version 6.14 Theory Manual.

[jrpad7ec3bib7] DJurović B, Radjen S, Radenković M, Dragović T, Tatomirović Z, Ivanković N, Vukmirović D, Dugonjić S (2016). Chernobyl and Fukushima nuclear accidents: what have we learned and what have we done?. Vojnosanit Pregl.

[jrpad7ec3bib8] Farfán E B, Han E Y, Bolch W E, Huh C, Huston T E, Bolch Jr W E (2004). A revised stylized model of the adult extrathoracic and thoracic airways for use with the ICRP-66 human respiratory tract model. Health Phys..

[jrpad7ec3bib9] Gregoratto D, Bailey M R, Marsh J W (2010). Modelling particle retention in the alveolar–interstitial region of the human lungs. J. Radiol. Prot..

[jrpad7ec3bib10] Hamilton R I, Papadopoulos P N (2023). Using SHAP values and machine learning to understand trends in the transient stability limit. IEEE Trans. Power Syst..

[jrpad7ec3bib11] Huston T E (1995). Quantifying Uncertainties in Lung Dosimetry with Application to Plutonium Oxide Aerosols.

[jrpad7ec3bib12] Huston T E, Farfán E B, Bolch W E, Bolch W E (2003). Influences of parameter uncertainties within the ICRP-66 respiratory tract model: a parameter sensitivity analysis. Health Phys..

[jrpad7ec3bib13] IAEA (1989). Evaluating the Reliability of Predictions Made Using Environmental Transfer Models.

[jrpad7ec3bib14] ICRP (1991). 1990 Recommendations of the International Commission on Radiological Protection. ICRP Publication 60. Ann. ICRP.

[jrpad7ec3bib15] ICRP (1994). International Commission on Radiological Protection Publication 66: Human Respiratory Tract Model for Radiological Protection.

[jrpad7ec3bib16] ICRP (2002). Basic anatomical and physiological data for use in radiological protection: reference values: ICRP Publication 89. Ann. ICRP.

[jrpad7ec3bib17] ICRP (2007). International Commission on Radiological Protection Publication 103. Ann. ICRP.

[jrpad7ec3bib18] ICRP (2015). Occupational Intakes of Radionuclides, Part 1: ICRP Publication 130. Ann. ICRP.

[jrpad7ec3bib19] ICRP (2017). Occupational intakes of radionuclides: part 3. ICRP Publication 137. Ann. ICRP.

[jrpad7ec3bib20] Iman R L, Shortencarier M J, Johnson J D (1985). FORTRAN 77 program and user’s guide for the calculation of partial correlation and standardized regression coefficients.

[jrpad7ec3bib21] Klumpp J, Bertelli L (2017). KDEP: a resource for calculating particle deposition in the respiratory tract. Health Phys..

[jrpad7ec3bib22] Leggett R W (2001). Reliability of the ICRP’s dose coefficient for members of the Public. 1. Sources of uncertainty in the biokinetic models. Radiat. Prot. Dosim..

[jrpad7ec3bib23] Li W B (2018). Internal dosimetry―a review of progress. Jpn. J. Health Phys..

[jrpad7ec3bib24] Lundberg S M, Erion G, Chen H, DeGrave A, Prutkin J M, Nair B, Katz R, Himmelfarb J, Bansal N, Lee S-I (2020). From local explanations to global understanding with explainable AI for trees. Nat. Mach. Intell..

[jrpad7ec3bib25] Makumbi T, Breustedt B, Raskob W (2024). Parameter uncertainty analysis of the equivalent lung dose coefficient for the intake of radon in mines: a review. J. Environ. Radioact..

[jrpad7ec3bib26] Marcus C S, Siegel J A, Sparks R B (2006). Medical Management of Internally Radiocontaminated Patients.

[jrpad7ec3bib27] Marsh J W, Birchall A (2009). Uncertainty Analysis of the Absorbed Dose to Regions of the Lung per Unit Exposure to Radon Progeny in a Mine.

[jrpad7ec3bib28] Massey F J (1951). The Kolmogorov-Smirnov test for goodness of fit. J. Am. Stat. Assoc..

[jrpad7ec3bib29] Mate-Kole E M, Dewji S A (2024). Mathematical complexities in radionuclide metabolic modeling: a review of ordinary differential equation kinetics solvers in biokinetic modeling. J. Radiol. Prot..

[jrpad7ec3bib30] Mate-Kole E M, Margot D, Dewji S A (2023). Mathematical solutions in internal dose assessment: a comparison of python-based differential equation solvers in biokinetic modeling. J. Radiol. Prot..

[jrpad7ec3bib31] McKay M D (1992). Latin hypercube sampling as a tool in uncertainty analysis of computer models.

[jrpad7ec3bib32] McKay M D, Beckman R J (2000). A comparison of three methods for selecting values of input variables in the analysis of output from a computer code. Technometrics A.

[jrpad7ec3bib33] NCRP (1998). National council on radiation protection and measurements operational safety program.

[jrpad7ec3bib34] NCRP (2019). National council on radiation protection and measurements medical radiation exposure of patients in the united states.

[jrpad7ec3bib35] Pan P, Ungar R K (2012). Nuclear event zero-time calculation and uncertainty evaluation. J. Environ. Radioact..

[jrpad7ec3bib36] Paquet F (2022). Internal dosimetry: state of the art and research needed. J. Radiol. Prot. Res..

[jrpad7ec3bib37] Pedregosa F (2011). Scikit-learn: machine learning in Python. J. Mach. Learn. Res..

[jrpad7ec3bib38] Puncher M (2014). Assessing the reliability of dose coefficients for ingestion and inhalation of 226Ra and 90Sr by members of the public. Radiat. Prot. Dosim..

[jrpad7ec3bib39] Puncher M, Burt G (2013). The reliability of dose coefficients for inhalation and ingestion of uranium by members of the public. Radiat. Prot. Dosim..

[jrpad7ec3bib40] Sjögren R, Svensson D (2018). pydoe2: an experimental design package for python. GitHub Repository. https://github.com/clicumu/pyDOE2.

[jrpad7ec3bib41] Stein M (1987). Large sample properties of simulations using Latin hypercube sampling. Technometrics.

[jrpad7ec3bib42] Sulaiman S N A, Mohamed F, Ab Rahim A N (2018). Radioactive release during nuclear accidents in Chernobyl and Fukushima. IOP Conf. Ser.: Mater. Sci. Eng..

[jrpad7ec3bib43] USEPA (2024). Radionuclide Basics: Iodine.

[jrpad7ec3bib44] Virtanen P (2020). SciPy 1.0: fundamental algorithms for scientific computing in Python. Nat. Methods.

[jrpad7ec3bib45] Zanzonico P B (2000). Internal radionuclide radiation dosimetry: a review of basic concepts and recent developments. J. Nucl. Med..

